# Supplementary material for: Imination of Microporous Chitosan Fibers—A Route to Biomaterials with “On Demand” Antimicrobial Activity and Biodegradation for Wound Dressings
Source: Pharmaceutics. 2022 Jan 4;14(1):117. doi: 10.3390/pharmaceutics14010117 (PMC8777909; doi:10.3390/pharmaceutics14010117)
Supplement: Supplementary file 1 [file pharmaceutics-14-00117-s001.zip › pharmaceutics-1502080-supplementary.pdf]

pharmaceutics

Supplementary Materials: Imination of Microporous Chitosan Fibers—A Route to Biomaterials with "on Demand" Antimicrobial Activity and Biodegradation for Wound Dressings

Alexandru Anisiei, Irina Rosca, Andreea-Isabela Sandu, Adrian Bele, Xinjian Cheng and Luminita Marin *

Determination of the acetylation degree of chitosan

The degree of deacetylation (**DA**) of chitosan was determined by ^{1}H-NMR spectroscopy and was calculated according to the Equation (S1) [1]:

$$DA\ (\%) = [(1/3\ I_{CH_3})/(1/6\ I_{H_2\text{-}H_6})] \times 100 \qquad (S1)$$

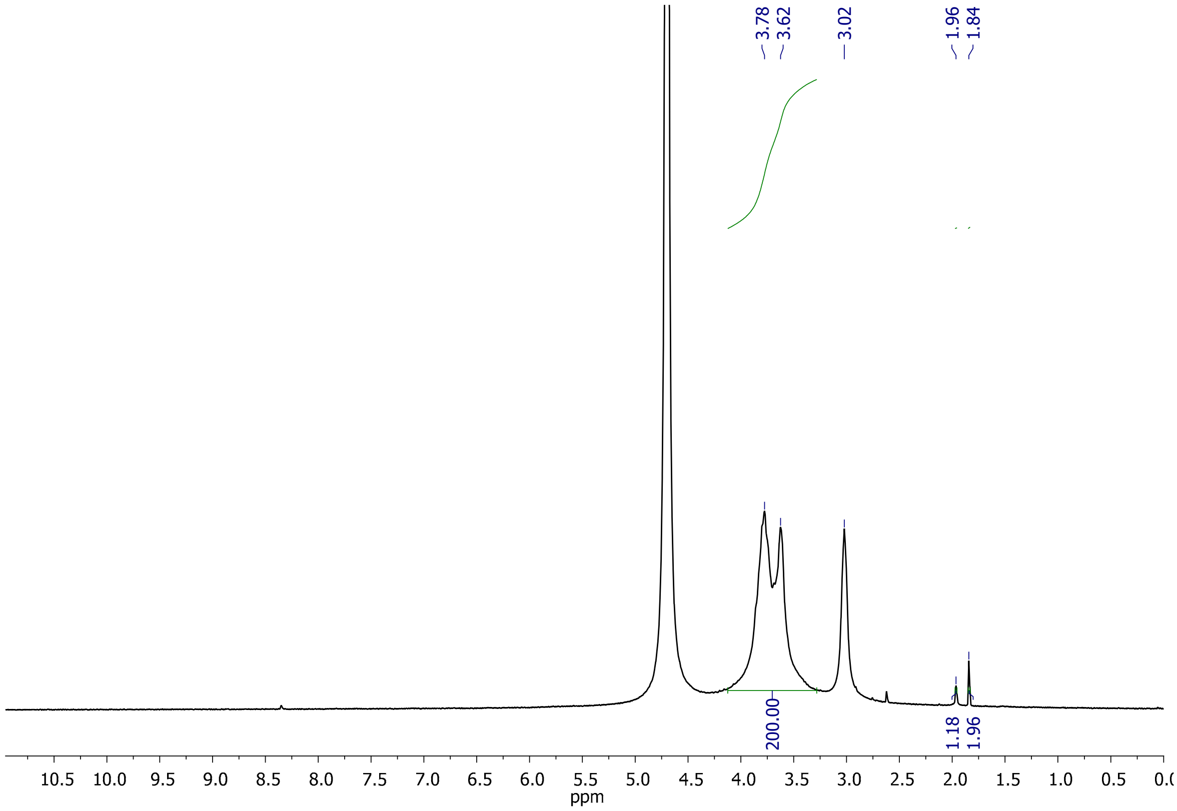

Figure S1. ^{1}H-NMR of chitosan in 2% acetic acid in deuterated water: 4.3-3.2 (H_2-H_6), 1.97, 1.7 (s, 3H, -CH_3).

Determination of the molecular weight of chitosan

The molecular weight of chitosan was determined by viscosity measurements, using a Ubbelohde viscometer. A stock solution of concentration 0.5 g/dL chitosan in a mixture of 0.3M acetic acid/0.2M sodium acetate (1/1 V/V) was prepared, and used to obtain four solutions of concentrations in the range 0.02–0.08 g/dL, by dilution with the same solvent. The flow-time through a 0 capillary, at 25 °C was

measured for each solution, including the solvent mixture as reference. The reduced viscosity of the solutions was calculated using the Equations (S2)–(S4), and then it a graph of the reduced viscosity as a function of chitosan solution concentration was drawn. The correlation between the obtained linear equation and the molecular weight of chitosan was achieved using the Mark–Houwink–Sakurada Equation (S5) [2].

$$\eta_{rel} = t_i/t_0 \qquad \text{(S2)}$$

$$\eta_{sp} = \eta_{rel} - 1 \qquad \text{(S3)}$$

$$\eta_{red} = \eta_{sp}/\text{concentration} \qquad \text{(S4)}$$

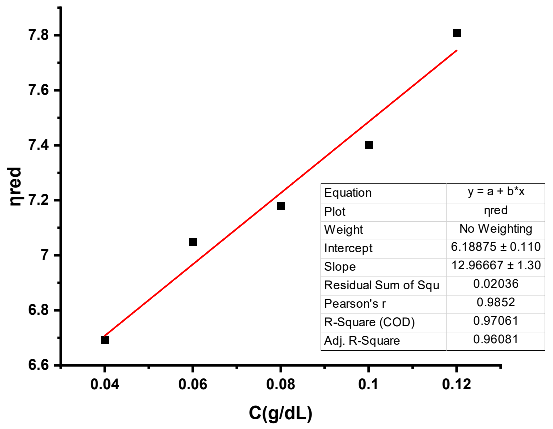

Figure S2. Graphical representation of the reduced viscosity as a function of the chitosan solution concentration.

$$[\eta] = k * Mv^{a} \qquad \text{(S5)}$$

In Equation (S5), the [η] represents the intrinsic viscosity determined from the graphical representation whereas "k" and "a" are the constants of the solvent mixture at 25 °C, characteristic for a deacetylation degree (DD) = 97% and are equal to: $k = 74 \times 10^{-5}$ g/dL and a = 0.76.

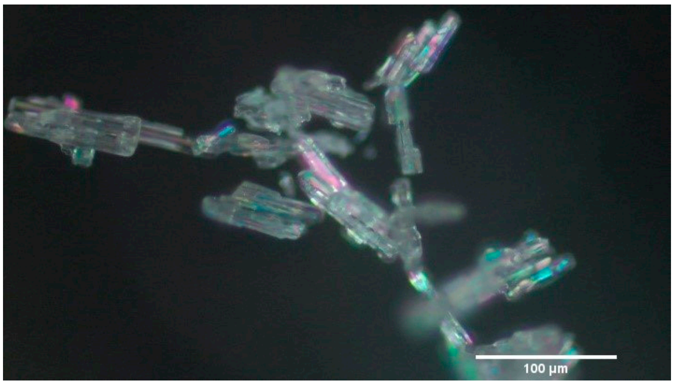

Figure S3. POM images of the 2-formylphenylboronic acid crystals purified by column chromatography.

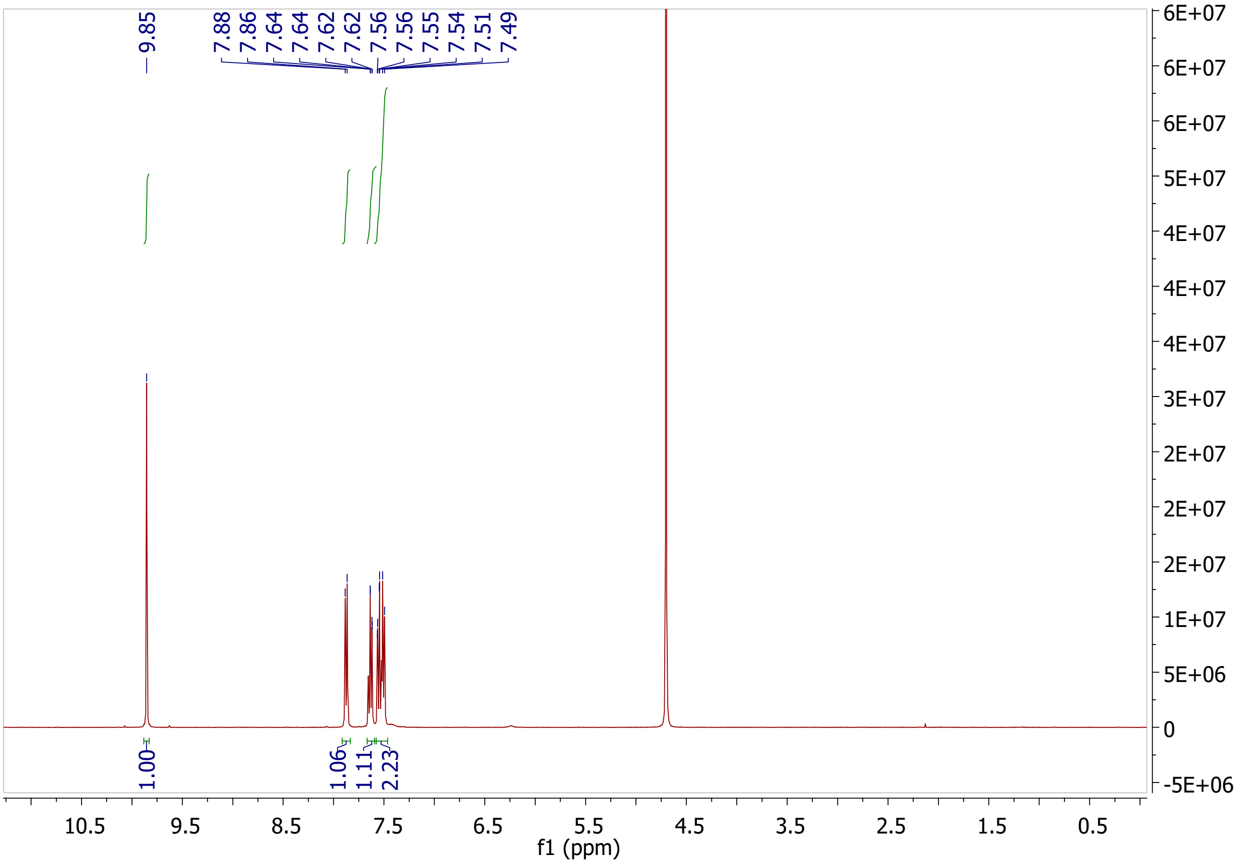

Figure S4. ^{1}H-NMR spectrum of the 2-formylphenylboronic acid purified by column chromatography.

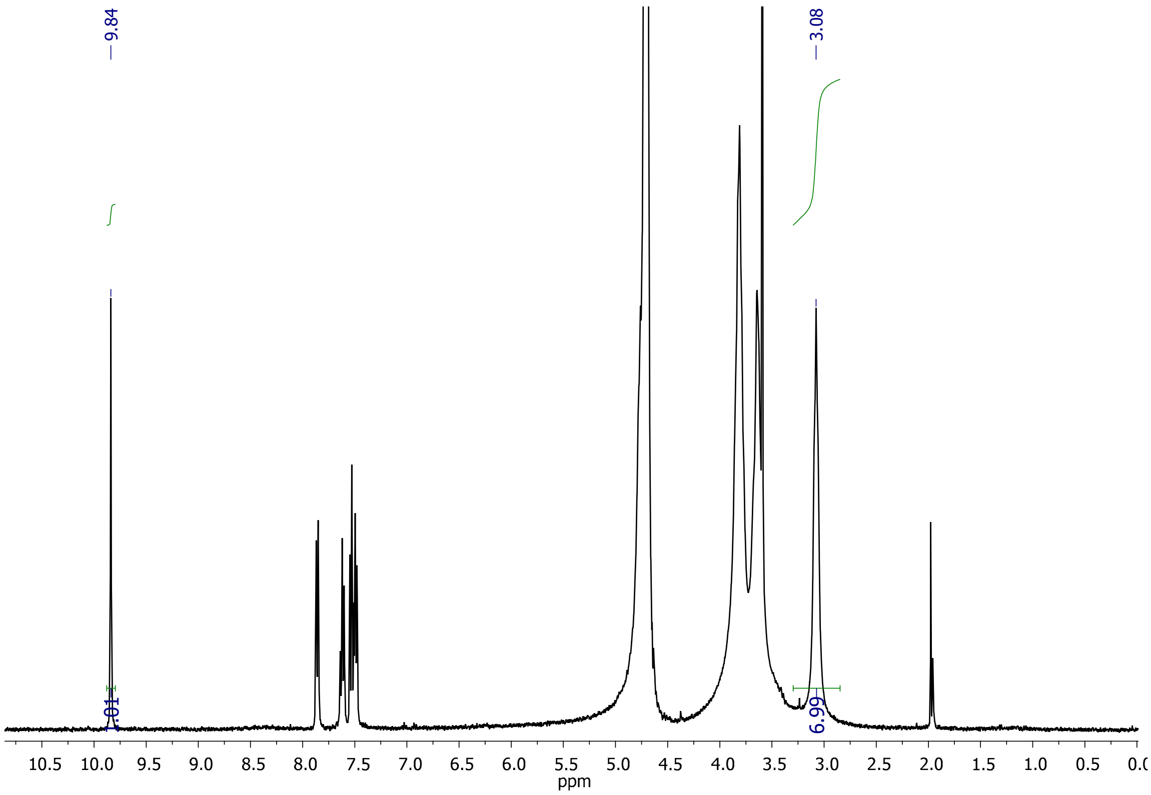

(a) BC1

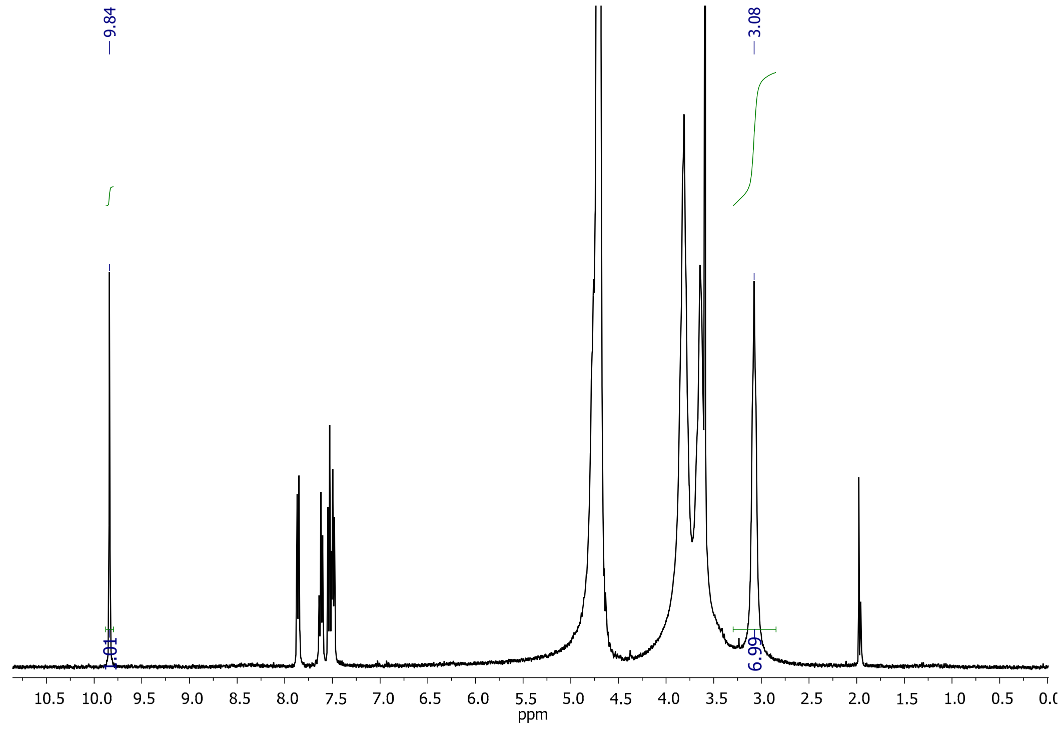

9.84
3.08
1.01
6.99
10.5 10.0 9.5 9.0 8.5 8.0 7.5 7.0 6.5 6.0 5.5 5.0 4.5 4.0 3.5 3.0 2.5 2.0 1.5 1.0 0.5
ppm

(b) BC1R

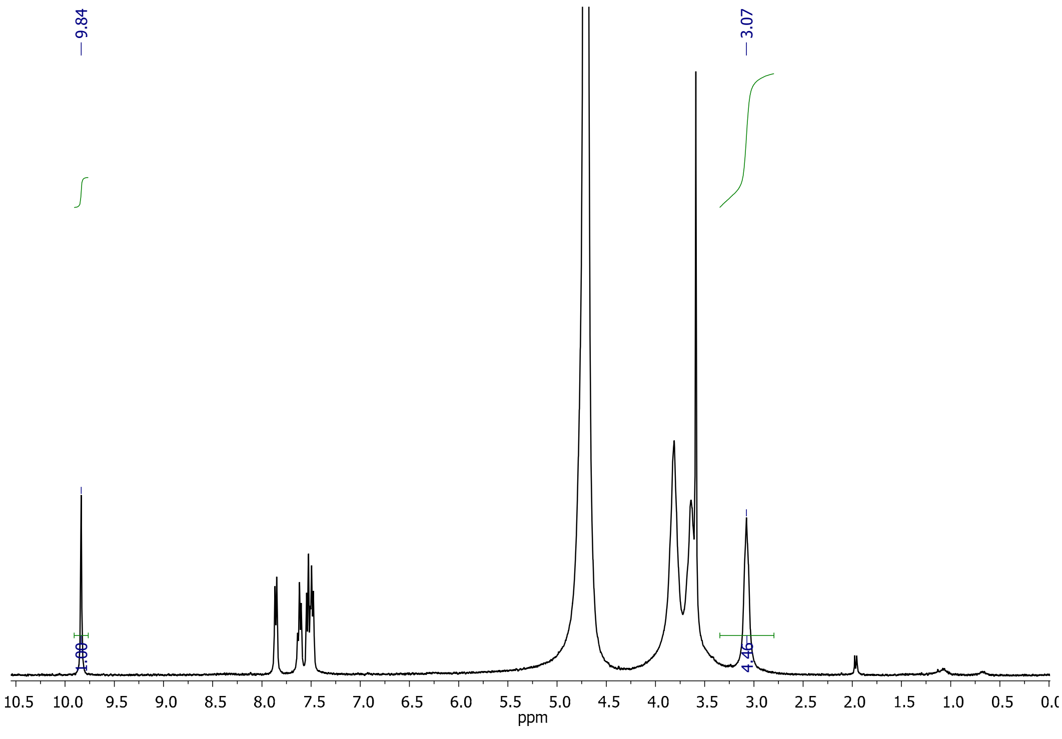

9.84
3.07
1.00
4.46
10.5 10.0 9.5 9.0 8.5 8.0 7.5 7.0 6.5 6.0 5.5 5.0 4.5 4.0 3.5 3.0 2.5 2.0 1.5 1.0 0.5
ppm

(c) BC2

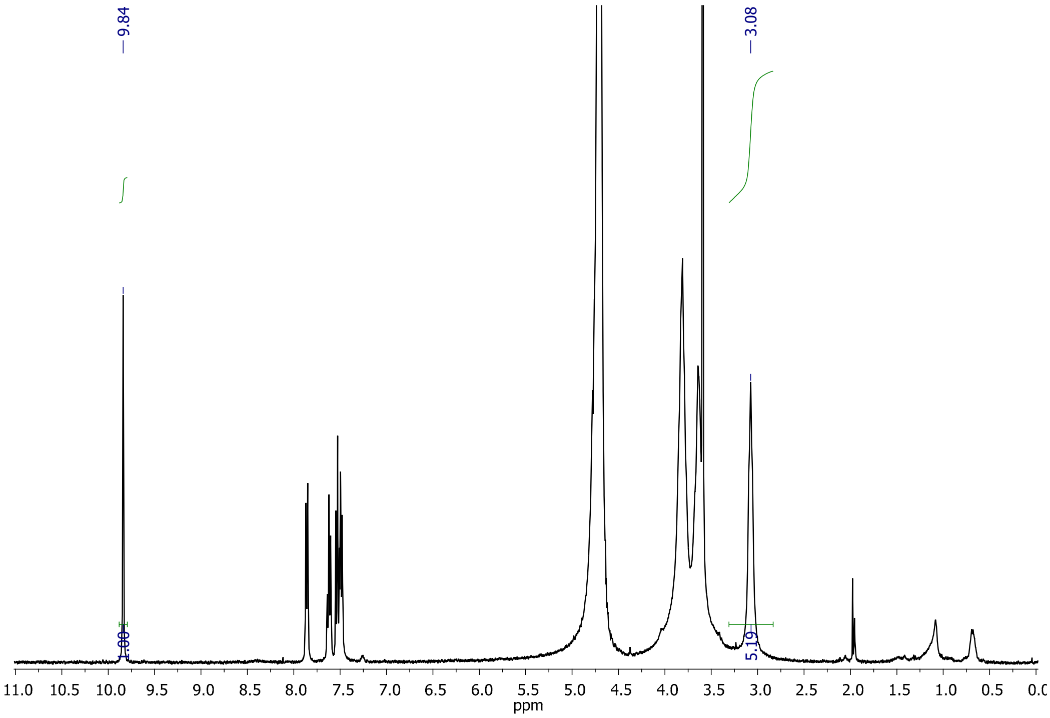

(d) BC4

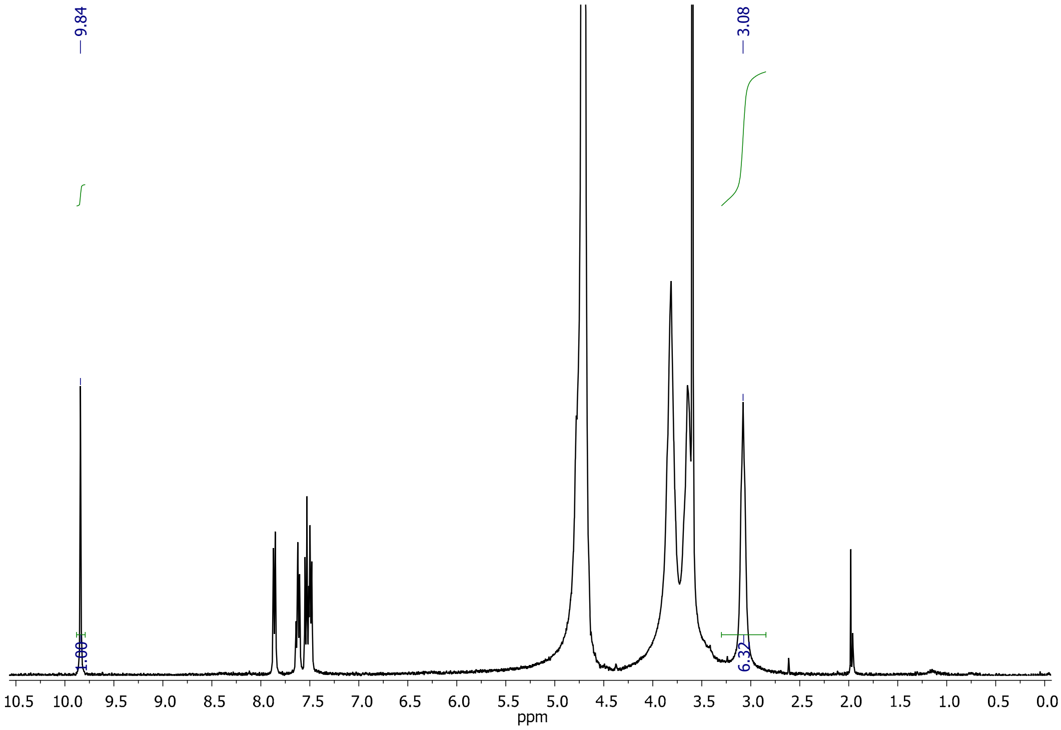

(e) BC6

Figure S5. ^{1}H-NMR spectra of the fibers functionalized with 2-formylphenylboronic.

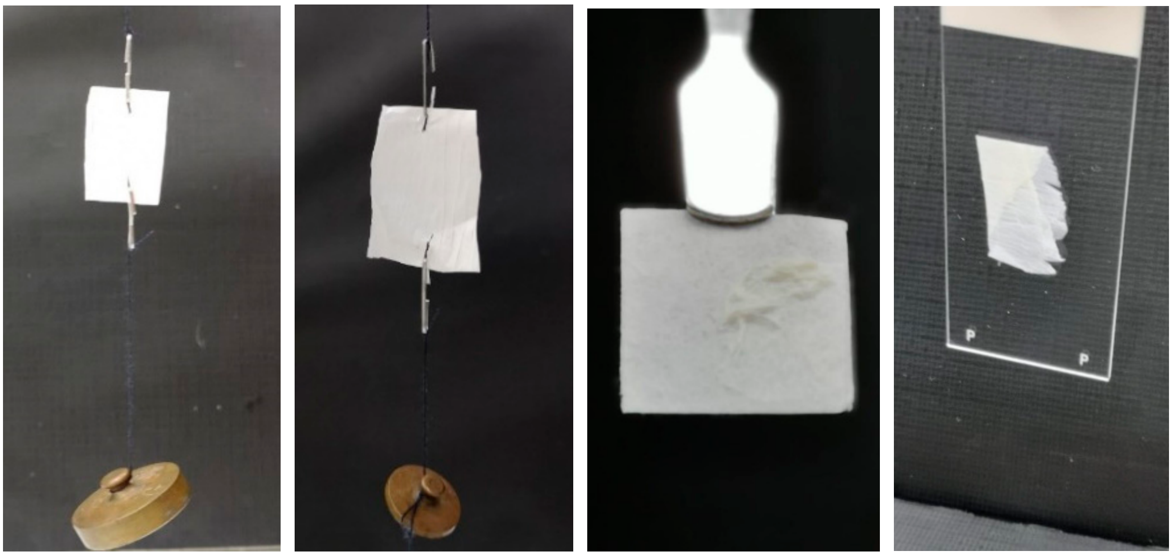

(**a**) (**b**) (**c**) (**d**)

Figure S6. Images of the (**a**) **CS** and (**b**) **BC6** fibers supporting balance weights of 50 g and images of **BC6** sample adhered on the surface of (**c**) a filter paper and (**d**) a glass lamellae moisture in PBS buffer.

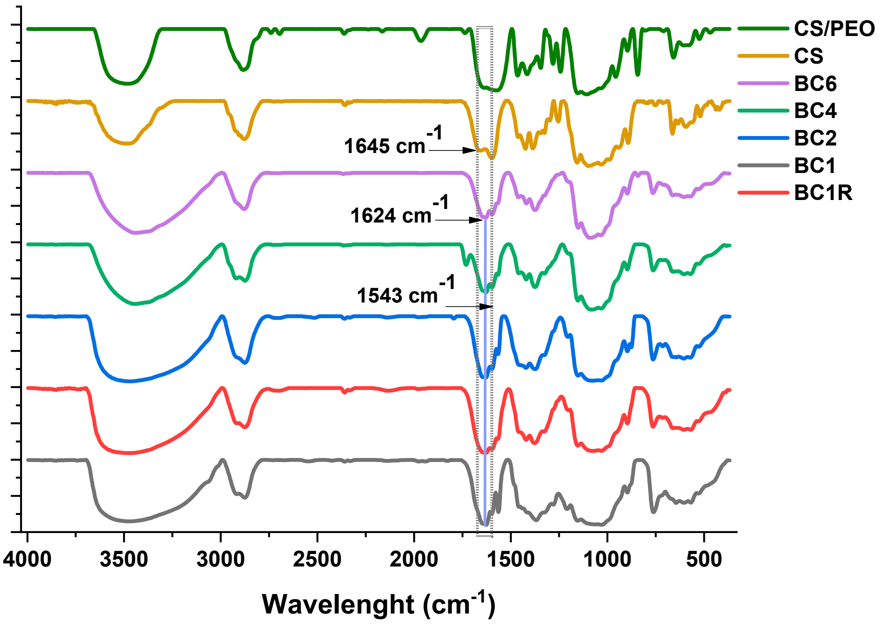

Figure S7. FTIR spectra of the **CS/PEO**, **CS** and imino-chitosan fibers.

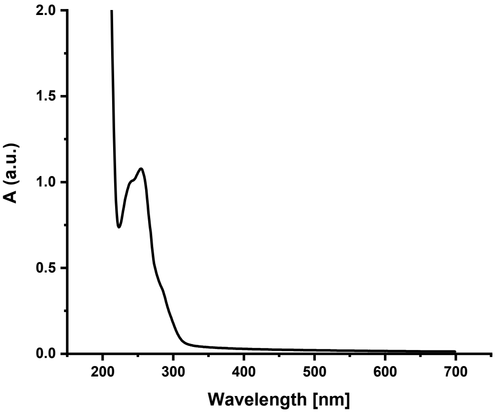

(a) 1h, A=1,078

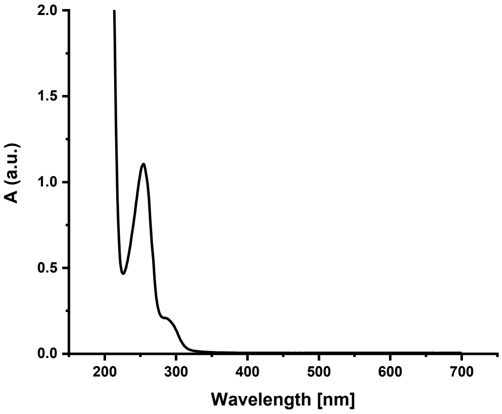

(b) 6h, A=1,104

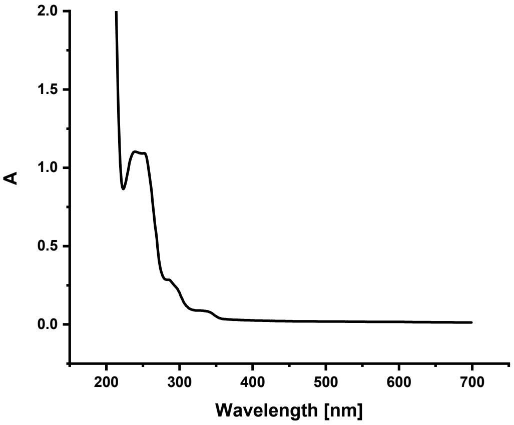

(c) 24 h, A=1,068

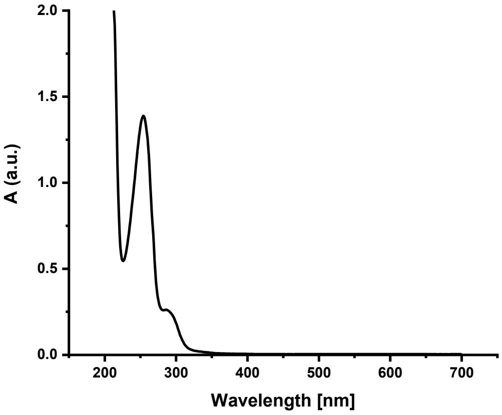

(d) 24h, A=1.386

Figure S8. UV-vis spectra of the supernatant from the **BC1R** fibers incubated in phosphate buffer (PBS), withdrawn at certain moments and replenished with fresh PBS (**a**–**c**), or when the supernatant was withdrawn after 24 h (**d**). The moment time when the supernatant was withdrawn and the absorbance were given under the graphs. It can be observed that the absorbance has close values, no matter the removal time of the supernatant, indicating that the aldehyde release depends in principal by the imination equilibrium.

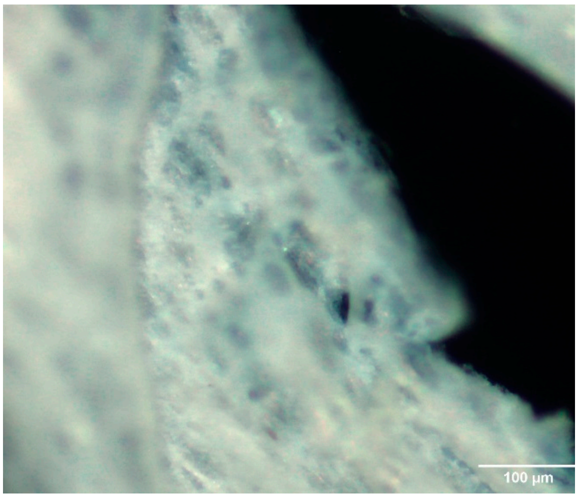

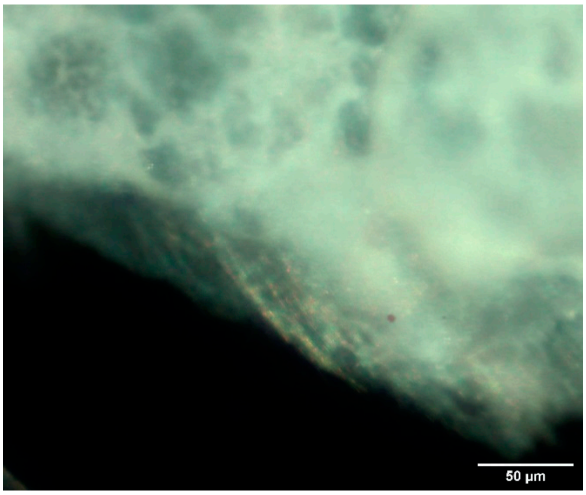

(a) CS-PEG **(b) CS**

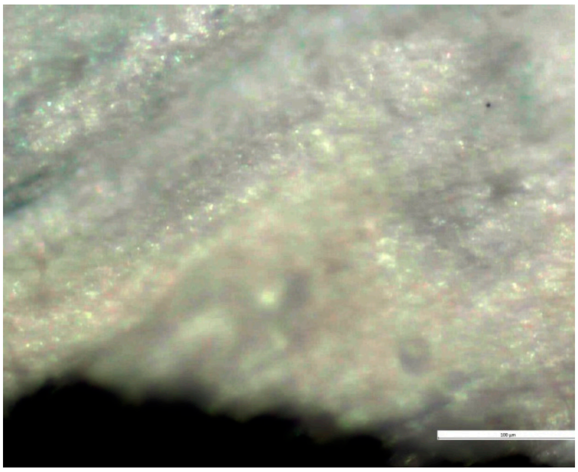

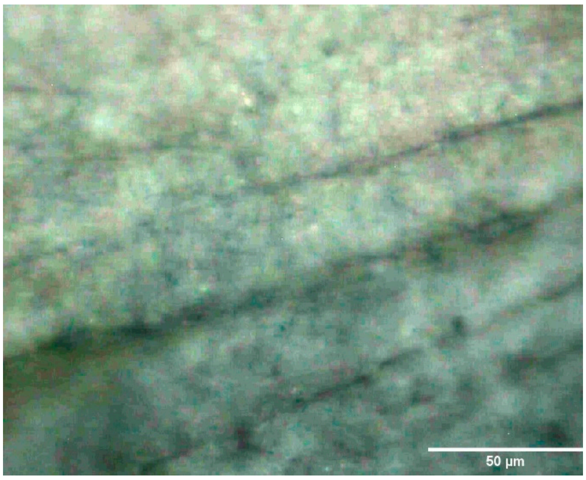

(c) BC1 **(d) BC2**

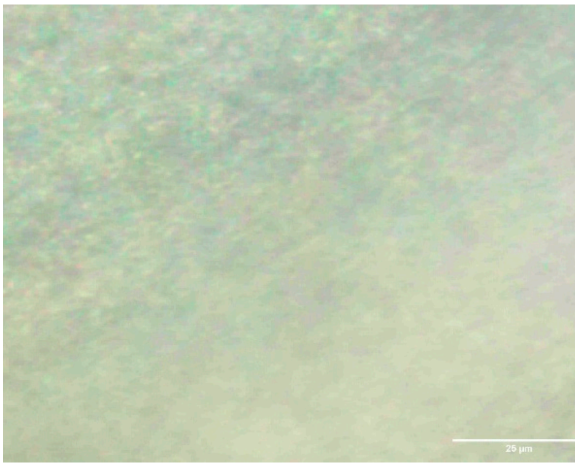

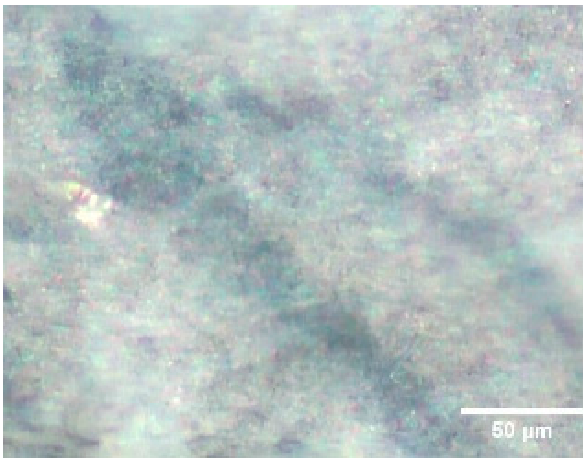

(e) BC4 **(f) BC6**

Figure S9. POM microphotographs of the studied samples.

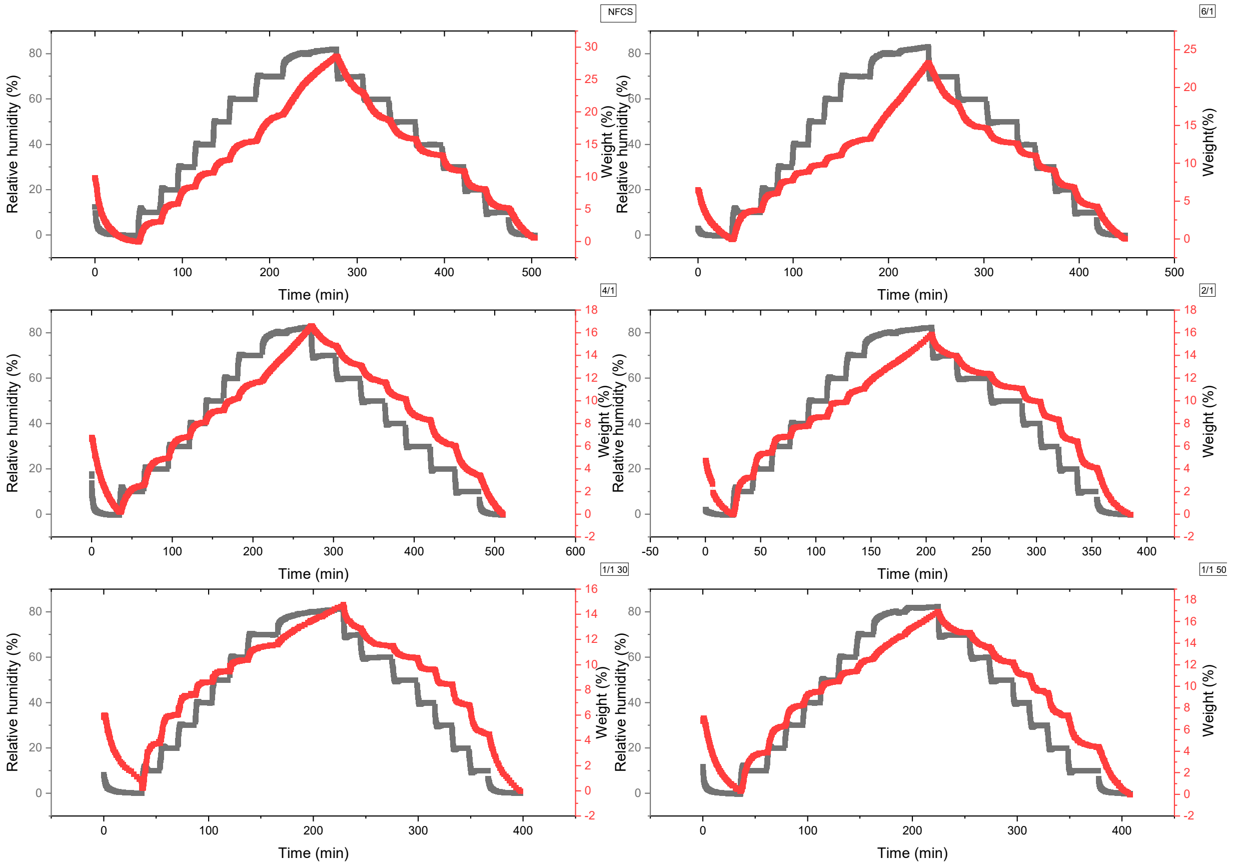

Figure S10. The kinetics of water vapour sorption uptake (Grey colour was used for the steps of increasing the external vaporized water content; Red colour was used for the successive mass uptake that is a response to the steps of increasing of the external water content, the curves being at equilibrium point of each step when reach the saturation point.).

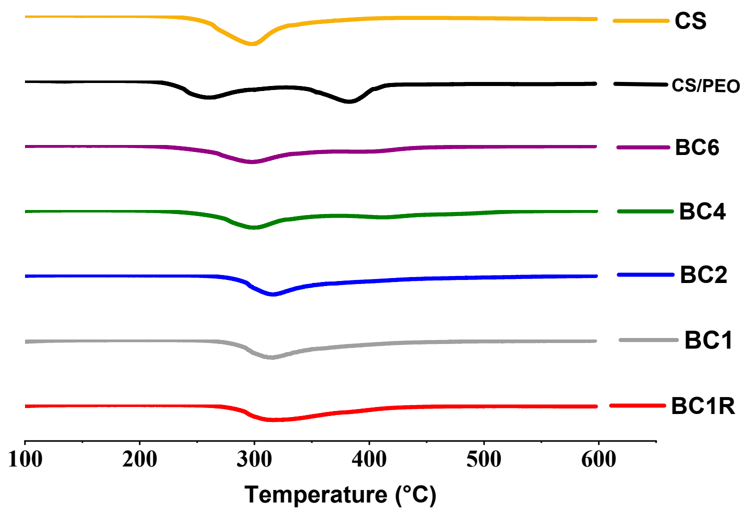

Figure S11. DTG curves of the chitosan-based nanofibers.

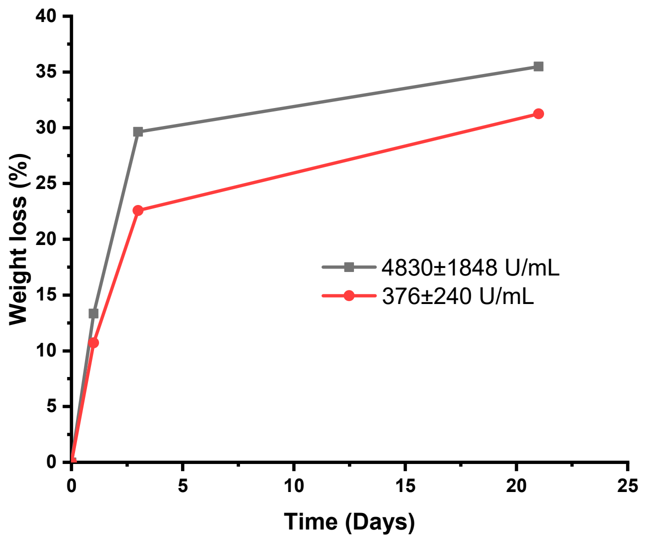

Figure S12. Representative curves for kinetics of enzymatic biodegradation of BC1R in lysozyme in PBS of pH = 8.5 characteristic for non-infected (376 ± 240 U/mL) and infected (4830 ± 1848 U/mL) wounds during 21 days.

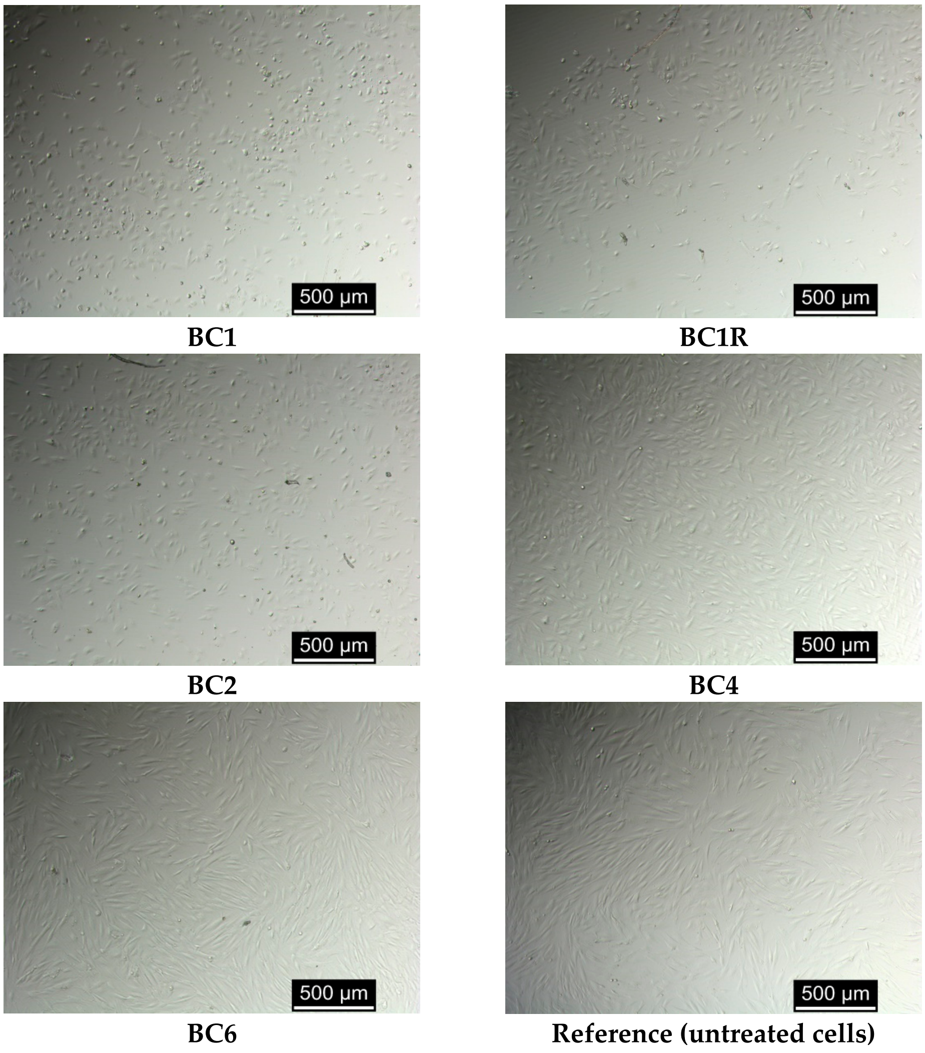

Figure S13. Brighfield microscopy images of the cells exposed for 24 h to fiber mats.

References

1. Hirai, A.; Odani, H.; Nakajima, A. Determination of degree of deacetylation of chitosan by 1H NMR spectroscopy. *Polym. Bull.* **1991**, *26*, 87–94. https://doi.org/10.1007/BF00299352.
2. Kasaai, M.R. Calculation of Mark–Houwink–Sakurada (MHS) equation viscometric constants for chitosan in any solvent–temperature system using experimental reported viscometric constants data. *Carbohydr. Polym.* **2007**, *68*, 477–488. https://doi.org/10.1016/j.carbpol.2006.11.006.
